# Genome sequence analysis of *Helicobacter pylori *strains associated with gastric ulceration and gastric cancer

**DOI:** 10.1186/1471-2164-10-3

**Published:** 2009-01-05

**Authors:** Mark S McClain, Carrie L Shaffer, Dawn A Israel, Richard M Peek, Timothy L Cover

**Affiliations:** 1 Department of Medicine, Vanderbilt University School of Medicine, Nashville, TN 37232-2605, USA; 2 Department of Microbiology and Immunology, Vanderbilt University School of Medicine, Nashville, TN 37232-2605, USA; 3 Veterans Affairs Tennessee Valley Healthcare System, Nashville, TN 37212, USA

## Abstract

**Background:**

Persistent colonization of the human stomach by *Helicobacter pylori *is associated with asymptomatic gastric inflammation (gastritis) and an increased risk of duodenal ulceration, gastric ulceration, and non-cardia gastric cancer. In previous studies, the genome sequences of *H. pylori *strains from patients with gastritis or duodenal ulcer disease have been analyzed. In this study, we analyzed the genome sequences of an *H. pylori *strain (98-10) isolated from a patient with gastric cancer and an *H. pylori *strain (B128) isolated from a patient with gastric ulcer disease.

**Results:**

Based on multilocus sequence typing, strain 98-10 was most closely related to *H. pylori *strains of East Asian origin and strain B128 was most closely related to strains of European origin. Strain 98-10 contained multiple features characteristic of East Asian strains, including a type s1c *vacA *allele and a *cagA *allele encoding an EPIYA-D tyrosine phosphorylation motif. A core genome of 1237 genes was present in all five strains for which genome sequences were available. Among the 1237 core genes, a subset of alleles was highly divergent in the East Asian strain 98-10, encoding proteins that exhibited <90% amino acid sequence identity compared to corresponding proteins in the other four strains. Unique strain-specific genes were identified in each of the newly sequenced strains, and a set of strain-specific genes was shared among *H. pylori *strains associated with gastric cancer or premalignant gastric lesions.

**Conclusion:**

These data provide insight into the diversity that exists among *H. pylori *strains from diverse clinical and geographic origins. Highly divergent alleles and strain-specific genes identified in this study may represent useful biomarkers for analyzing geographic partitioning of *H. pylori *and for identifying strains capable of inducing malignant or premalignant gastric lesions.

## Background

*Helicobacter pylori *is a Gram-negative spiral-shaped bacterium that persistently colonizes the human stomach [1]. Persistent *H. pylori *colonization of the human stomach is a risk factor for several diseases, including non-cardia gastric adenocarcinoma, gastric lymphoma, and peptic ulceration [1,2]. The incidence of these diseases varies considerably throughout the world. For example, the incidence of gastric adenocarcinoma is substantially higher in East Asia, Central America, and South America than in most other parts of the world [3].

*H. pylori *isolates from unrelated humans exhibit a high level of genetic diversity [4,5]. Genetic variation is readily detectable by analyzing the nucleotide sequences of individual genes in different *H. pylori *strains [6]. *H. pylori *allelic diversity is probably the consequence of multiple factors, including a high rate of mutation, a high rate of intraspecies genetic recombination, and a long evolutionary history of the species [4,7]. Corresponding alleles in different *H. pylori *strains typically are 92 to 99% identical in nucleotide sequences [4,6], but several *H. pylori *genes exhibit a much higher level of genetic diversity [8,9].

Further analyses have shown that there is geographic variation among *H. pylori *strains [10-16]. Based on multilocus sequence analysis of a panel of 370 *H. pylori *strains isolated from humans in different parts of the world, seven populations of strains with distinct geographic distributions have been identified [17]. These *H. pylori *populations reflect the migration of humans from Africa to other parts of the world over a time period estimated to be approximately 58,000 years [12]. Geographic differences among *H. pylori *strains could potentially be a factor that helps to explain the varying incidence of *H. pylori*-associated diseases in various parts of the world.

In addition to variation among *H. pylori *strains in the sequences of individual genes, there is considerable variation among strains in gene content. One study analyzed genomic DNA from 56 different *H. pylori *strains using array hybridization methods and identified 1150 genes that were present in all of the strains tested (thus representing a "core" genome) [18]. Among 1531 genes analyzed, 25% were absent from at least one of the 56 *H. pylori *strains. It was predicted that the *H. pylori *core genome would consist of 1,111 genes if a much larger set of isolates were tested [18]. Other studies have reported the existence of core genomes comprising 1091 or 1281 genes, based on DNA array analysis of 34 or 15 *H. pylori *strains, respectively [19,20]. One study reported that the phylogeny of *H. pylori *strains based on MLST analysis was substantially different from the phylogeny of *H. pylori *strains based on analysis of gene content [18].

One of the most striking differences in gene content among *H. pylori *strains is the presence or absence of a 40-kb region of chromosomal DNA known as the *cag *pathogenicity island (PAI) [8,21-24]. In the United States and Europe, about 50–60% of *H. pylori *strains contain the *cag *PAI and the remaining strains lack this region of the chromosome [8,21-24]. In many other parts of the world, including East Asia, nearly all *H*. *pylori *strains contain the *cag *PAI [15,25,26]. The *H. pylori cag *PAI encodes an effector protein, CagA, and a type IV secretion apparatus that translocates CagA into gastric epithelial cells [27]. *H. pylori *strains harboring the *cag *PAI are associated with an increased risk of non-cardia gastric cancer or peptic ulcer disease compared to strains that lack the *cag *PAI [21,28]. The correlation between these diseases and presence of the *cag *PAI provides an example of how the clinical outcome of *H. pylori *infection is determined in part by genetic characteristics of the strains with which a person is infected.

In previous studies, the complete genomes of three *H. pylori *strains have been analyzed [29-31]. These three *H. pylori *strains were isolated from patients who had gastritis, atrophic gastritis, or duodenal ulcer disease. In the current study, we sought to analyze genetic features of *H. pylori *strains isolated from patients with two different *H. pylori*-associated diseases: gastric ulcer and gastric cancer. For this analysis, we selected a gastric ulcer strain (B128) that readily colonizes the stomachs of mice and Mongolian gerbils. This strain is of particular interest because an animal-passaged derivative of strain B128 (strain 7.13) causes gastric cancer in a Mongolian gerbil model [32,33]. For an analysis of a gastric cancer-associated *H. pylori *strain, we selected strain 98-10, which was isolated from a gastric cancer patient in Japan [34], a country with a very high incidence of gastric cancer [3,35].

## Results

### General features of *H. pylori *genomes

Prior to the current study, the complete genome sequences of *H. pylori *strains isolated from patients with superficial gastritis, atrophic gastritis, or duodenal ulcer disease had been reported [29-31]. In the current study, we analyzed the genome sequences of an *H. pylori *strain (98-10) that was isolated from a patient with gastric cancer [34] and a strain (B128) that was isolated from a patient with gastric ulcer disease [32]. General features of the two genomes analyzed in the current study in comparison to three previously sequenced genomes are summarized in Table 1. To identify transposable genetic elements that might be present in the two newly sequenced genomes, the nucleotide sequences of each genome were used as queries to search an insertion sequence database . Strain 98-10 contained ORFs (HP9810_5g1 and HP9810_5g2) homologous to ORFs found in IS607 (accession number AF189015) [36]. Strain B128 contained ORFs (HPB128_26g16, HPB128_26g17, and HPB128_26g18) homologous to ORFs found in ISHp608 (accession number AF357224), but nucleotide insertions are predicted to disrupt the transposase gene in strain B128 [37]. IS607 and ISHp608 are not present in any of the three *H. pylori *strains for which genome sequences were previously available. A previous study reported that IS607 was detected in about 20% of *H. pylori *strains [36]. ISHp608 is nonrandomly distributed geographically among *H. pylori *strains, and this element was reported to be more abundant in strains from Peruvian patients with gastric cancer than in strains from Peruvian patients with gastritis only [37].

**Table 1:** Features of *H. pylori *genomes

	*H. pylori strain*				
	26695	J99	HPAG1	98-10	B128
Origin	U.K.	U.S.	Sweden	Japan	U.S.
Disease state^a^	Gastritis only	DU	AG	GC	GU
*cag *PAI	Yes	Yes	Yes	Yes	Yes
*vacA *genotype	s1a/m1	s1b/m1	s1b/m1	s1c/m1	s1a/m2^h^
Genome size (Mb)	1.67	1.64	1.61^b^	1.6^c^	1.6^c^
Total no. of ORFs	1564^d^	1491^e^	1544^f^	1527	1731
No. of strain-specific genes^g^	69	23	38	22	51

^a ^DU, duodenal ulcer; AG, atrophic gastritis; GC, gastric cancer; GU, gastric ulcer

^b ^Includes a 9.3 kb plasmid.

^c ^The genome size of strain 98-10 is based on analysis of 51 large contigs, as defined in Methods. The genome size of strain B128 is based on analysis of 73 large contigs.

^d ^The current analysis is based on data downloaded from TIGR, comprising 1564 ORFs. In contrast, a table on the TIGR website lists 1587 ORFs in strain 26695, and Genbank sequence files include 1566 ORFs from strain 26695.

^e ^Additional ORFs, not included in this total, were subsequently detected in strain J99 [43].

^f ^The HPAG1 chromosome contains 1,536 predicted protein-coding genes, and the remainder are contained on a plasmid.

^g ^Present in only one of five strains analyzed in this study.

^h ^*vacA *is truncated in strain B128.

### MLST analysis of *H. pylori *strains

**Figure 1:**
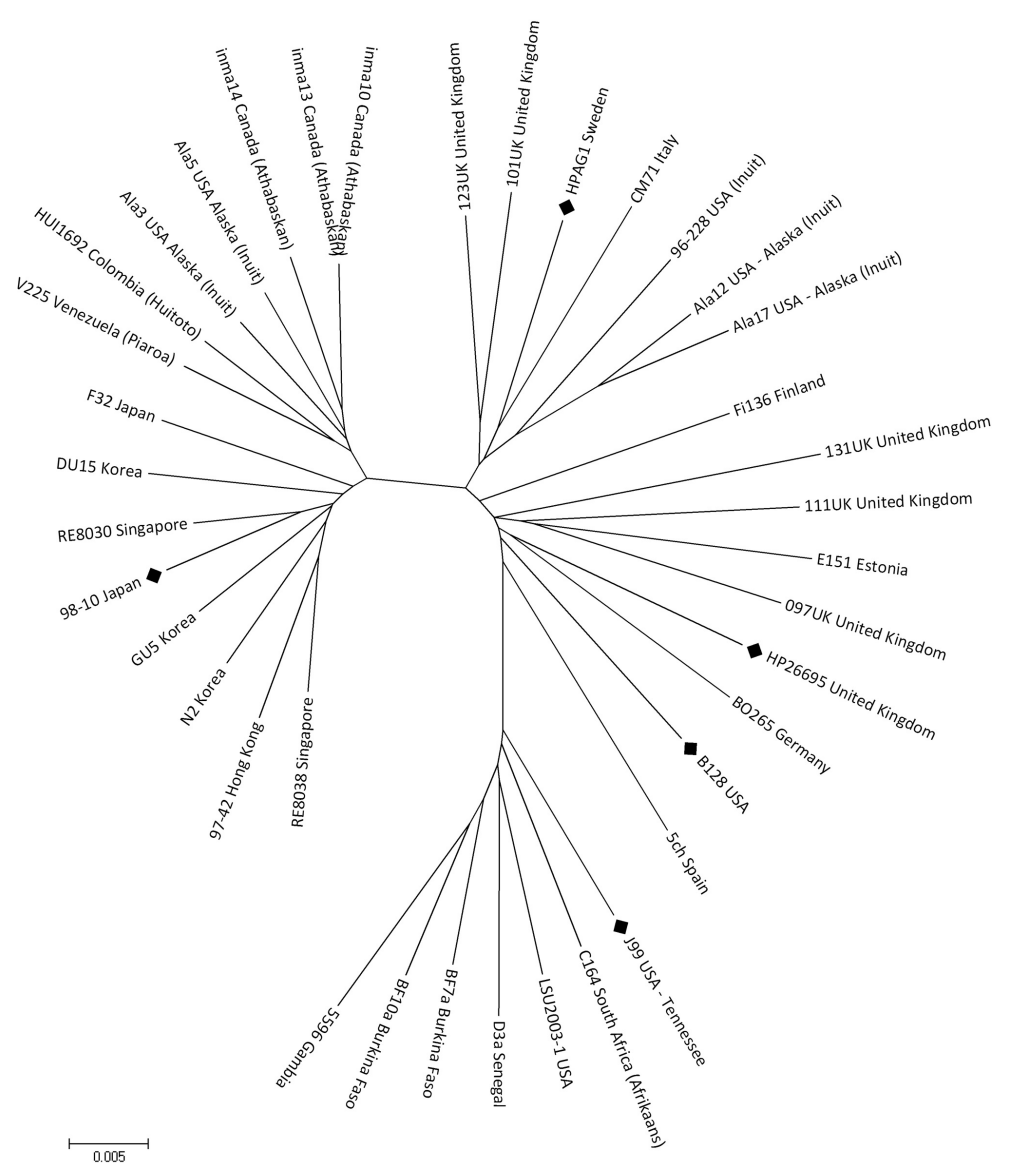
**Phylogenetic structure based on sequence analysis of 8 *H. pylori *core genes**. *H. pylori *strains analyzed in this figure include strains 98-10, B128, three strains for which genome sequences were previously determined (26695, J99, HPAG1), and representative strains isolated from patients in diverse geographic locations [18]. The figure lists the strain designations and the countries where strains were isolated. The nucleotide sequences of the concatenated MLST loci were aligned and compared, as described in Methods. All positions containing gaps and missing data were eliminated from the dataset. There were a total of 3041 positions in the final dataset. Neighbor-joining trees were constructed based on distances estimated by the Kimura 2-parameter model of nucleotide substitution [57,58]. The bootstrap consensus tree inferred from 1000 replicates is taken to represent the evolutionary history of the strains analyzed [59]. Branches corresponding to partitions reproduced in fewer than 50% bootstrap replicates are collapsed. The tree is drawn to scale, with the branch lengths in the same units as those of the evolutionary distances used to infer the phylogenetic tree. Phylogenetic analyses were conducted in MEGA4 [63]. Five *H. pylori *strains for which genome sequences were available are denoted by diamonds. Three main *H. pylori *population groups (East Asian, European, and West African) are identifiable.

In previous studies, MLST analysis has been used to classify *H. pylori *isolates into several haplogroups that have distinct geographic distributions [17]. To assign the two newly sequenced *H. pylori *strains to one of the previously described population clusters, we compared eight gene sequences from each strain to the corresponding sequences of 434 other *H. pylori *isolates, using an MLST database as described in the Methods. Based on this analysis, strain 98-10 was classified as a member of the East Asian population cluster and strain B128 was classified as a member of the European population cluster. A neighbor-joining tree depicting relationships of the two newly sequenced strains to representative reference strains isolated from diverse geographic locations is shown in Figure 1. The clustering depicted on this neighbor-joining tree accurately reflects the geographic origins of the reference strains, and is in agreement with previous assignments of the reference strains to distinct population groups [18]. In agreement with an earlier report [17], one of the previously sequenced *H. pylori *strains (J99) was most closely related to strains isolated in West Africa, and another (26695) was most closely related to strains isolated in Europe. A third *H. pylori *strain (HPAG1) analyzed in a prior study was closely related to strains isolated in Europe. Figure 1 illustrates that strain 98-10 is most closely related to strains of East Asian origin, and therefore, strain 98-10 belongs to a population cluster different from those of strains for which genome sequences were previously reported. Collectively, the genome sequences available for analysis represent three main geographic populations of *H. pylori *strains [European (26695, HPAG1, and B128), West African (J99), and East Asian (98-10)].

### Analysis of *cagA *and *vacA*

CagA and VacA are two important *H. pylori *virulence factors that are secreted by a type IV secretion pathway and a type V (autotransporter) secretion pathway, respectively [14,38]. Diversity in *cagA *and *vacA *genes has been investigated in detail in previous studies, and diversity in these genes provides a basis for typing *H. pylori *strains [8,13-15]. Therefore, we analyzed the *cagA *and *vacA *genes in each of the two newly sequenced strains.

When strain 98-10 was incubated with AGS gastric epithelial cells as described previously [39], CagA underwent tyrosine phosphorylation (data not shown), which indicates that this strain has a functional type IV secretion system for translocation of CagA into host cells [27]. The CagA protein encoded by strain 98-10 contains 3 EPIYA motifs (sites of tyrosine phosphorylation), which have been designated EPIYA-A, EPIYA-B, and EPIYA-D [14]. The presence of an EPIYA-D motif is characteristic of *H. pylori *strains isolated in East Asia [13,14]. Broth culture supernatant from strain 98-10 caused vacuolation of HeLa cells, indicating the presence of an active VacA toxin. This strain contains a type s1c/m1 *vacA *allele, a feature that is characteristic of *H. pylori *strains isolated in East Asia [15,40]. Identification of East Asian *cagA *and *vacA *motifs in strain 98-10 is consistent with the results of the MLST analysis, which classified strain 98-10 as a member of the East Asian population cluster of *H. pylori *strains.

Similar to strain 98-10, strain B128 has a functional type IV secretion system that can translocate CagA into gastric epithelial cells, and CagA subsequently undergoes tyrosine phosphorylation [41]. The CagA protein encoded by strain B128 contains two EPIYA motifs, designated EPIYA-A and EPIYA-C [14]. Strain B128 contains a type s1/m2 *vacA *allele, but a *vacA *mutation in this strain is predicted to prevent expression of a full-length VacA protein. The presence of the latter mutation was confirmed by nucleotide sequence analysis of a *vacA *fragment amplified by PCR. Immunoblot analysis using multiple anti-VacA antisera indicated that this strain did not produce a detectable VacA protein, and broth culture supernatant from this strain did not cause vacuolation of HeLa cells (data not shown).

### Characterization of the *H. pylori *core genome

**Figure 2:**
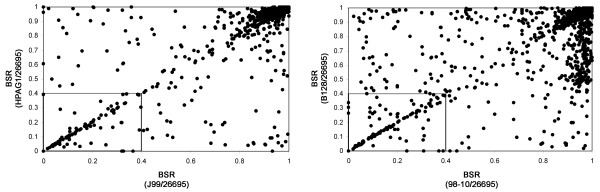
**Comparison of predicted proteomes by BLAST-score ratio (BSR) analysis**. The left panel shows a BSR analysis of proteins encoded by strain J99 and HPAG1, with strain 26695 as the reference strain. The right panel shows a BSR analysis of proteins encoded by strain 98-10 and B128, with strain 26695 as the reference strain. The BSR approach analyzes all proteins predicted to be encoded by three genomes, using a measure of similarity based on the ratio of BLAST scores, as described in the Methods. Proteins depicted within the box at the lower left corner (BSR <0.4) correspond to proteins present in the reference proteome (strain 26695) but absent from the two query proteomes. The upper right quadrant represents proteins conserved in all three proteomes.

Delineation of a *H. pylori *core genome (i.e. genes that are consistently present in all *H. pylori *isolates) is of interest, because many such genes are likely to be required for colonization of the human stomach. Based on the use of BLAST score ratio analysis as described in the Methods, we identified 1237 genes that were present in all 5 *H. pylori *genomes (Figure 2 and Additional file 1). In a previous study, 56 different *H. pylori *strains were analyzed by array methodology, and a core genome of 1150 genes was reported to be present in all 56 strains [18]. Among the 1150 genes reported to comprise the *H. pylori *core genome based on array analysis, 1094 were present in all 5 strains analyzed in the current study, as determined by sequence analysis. The list of core genes detected in all five strains by sequence analysis but not by array analysis includes >20 genes located within the *cag *PAI. Although the *cag *PAI is present in all 5 strains analyzed in the current study, this region of DNA is known to be absent from many *H. pylori *strains [24]. Five other clusters of contiguous genes (each with at least 4 genes per cluster) were present in all 5 sequenced strains, but were absent from the list of core genes identified by array analysis (HP0061–0065, HP0797–0800, HP1339–1343, HP1400–1403, and HP1455–1458) (Additional file 1). The differences in designation of core genes in the current study compared to previous studies can be attributed to numerous factors, including differences in the number of strains analyzed and differences in methodology for gene detection.

**Figure 3:**
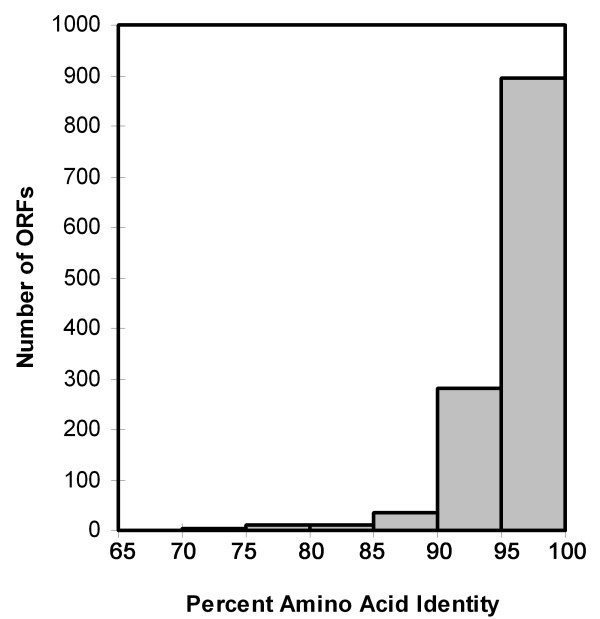
**Relatedness of core proteins predicted to be encoded by *H. pylori *strains 98-10 and 26695**. A set of 1237 genes present in all 5 *H. pylori *strains was identified, as described in the Methods. The deduced amino acid sequences of the corresponding proteins encoded by strain 98-10 were used to search a database of sequences from strain 26695 using FastA. The best match was identified, and the percent amino acid identity was calculated. The histogram shows the number of ORFs exhibiting the indicated level of amino acid identity.

An analysis of the 1237 core genes indicated that, in almost all cases, there were differences in the amino acid sequences of the proteins encoded by individual strains. Pairwise comparisons of proteins encoded by different strains indicated that the levels of relatedness ranged from 65% to 100% amino acid identity. A representative comparison of the core proteins encoded by two strains (98-10 and 26695) is shown in Figure 3. Only 11 genes were identified for which the amino acid sequences of encoded proteins were identical among all 5 strains. Seven of these 11 genes encoded ribosomal proteins; others encoded a translation initiation factor (IF-1), a lipoprotein (Lpp20), a flagellar basal body protein (FliE), and a protein of unknown function (HP0031).

### Analysis of divergent genes in an East Asian cancer-associated *H. pylori *strain

*H. pylori *strains isolated from unrelated humans exhibit allelic diversity (typically 92–99% nucleotide identity among corresponding alleles), which provides a basis for classification of strains into population clusters via MLST analysis. Several genes exhibit a substantially higher level of allelic diversity. For example, at least two genes (*cagA *and a *sel1 *homologue) are known to be markedly divergent in East Asian *H. pylori *strains compared to Western *H. pylori *strains [13,14,42]. We hypothesized that additional genes might be highly divergent in the East Asian strain 98-10 compared to the other 4 sequenced strains. To identify gene products encoded by the genome of 98-10 that are markedly divergent compared to products encoded by the other 4 genomes, we focused on analysis of the 1237 core genes that were present in all 5 sequenced strains. By using the approach described in Methods, we identified 8 gene products that were highly divergent in the East Asian strain compared to the other four strains (Table 2). These include CagA and a *sel1 *homologue, which were previously reported to be markedly divergent in East Asian strains compared to strains from other parts of the world [13,42]. The amino acid sequences of these divergent proteins encoded by the Japanese strain 98-10 were each <90% identical to sequences of corresponding proteins from the other four strains (Table 2). In each case, the divergent alleles in strain 98-10 and corresponding alleles in the other four strains were flanked by the same chromosomal genes.

**Table 2:** Highly divergent alleles in East Asian strain 98-10

Gene number (98-10)	Gene number (26695)	Description	% aa identity (98-10)^a^	% aa identity (non-98-10)^b^	% unique sites^c^
HP9810_903g20	HP0061^d^	Hypothetical	67	86	21
HP9810_889g5	HP0492^d^	*hpaA *homologue	72	92	21
HP9810_889g32	HP0519^d^	*sel1 *homologue	73	92	15
HP9810_905g13	HP0547	*cagA*	79	87	11
HP9810_868g41	HP0806^d^	Hypothetical	86	92	6
HP9810_899g75	HP1322^d^	Hypothetical	75	90	18
HP9810_899g76	HP1323^d^	Ribonuclease	88	92	6
HP9810_885g15	HP1524^d^	Hypothetical	80	95	13

^a ^The sequences of the indicated gene products in strain 98-10 were compared with corresponding sequences in each of the other 4 strains (26695, J99, HPAG1 and B128), and mean % amino acid identities were calculated as described in Methods.

^b ^The sequences of the indicated gene products in each strain were compared in all permutations, except that comparisons involving strain 98-10 were excluded from analysis. Mean % amino acid identities were calculated as described in Methods.

^c^Percentage of aligned sites in which the protein from strain 98-10 contained an amino acid different from the corresponding amino acids in proteins from 4 other strains.

^d^Reported to be a constituent of the *H. pylori *core genome, based on at least one array analysis [18-20].

As shown in Figure 1, strain J99 was most closely related to *H. pylori *strains isolated in West Africa, a population cluster different from those of the other strains for which genome sequences were available. Therefore, we hypothesized that specific genes might be highly divergent in the West African strain J99 compared to the other 4 sequenced strains. To identify such genes, we used the same approach as described above. Four unique highly divergent alleles were identified in strain J99 (Table 3), each encoding products that were <90% identical to corresponding proteins in the other four strains. Unique highly divergent alleles were not readily identifiable in strains 26695, HPAG1, or B128. A notable exception was the identification of a highly divergent *vacA *allele in strain B128 (gene HPB128_147g10). Identification of *vacA *as a divergent allele in strain B128 is attributable to the presence of an s1/m2 *vacA *allele in this strain and the presence of s1/m1 alleles in the four other strains; m1 and m2 forms of VacA typically exhibit only 60–70% amino acid identity within the mid-region of the protein [38].

**Table 3:** Highly divergent alleles in strain J99

Gene number (J99)	Gene number (26695)	Description	% aa identity (J99)^a^	% aa identity (non-J99)^b^	% unique sites^c^
jhp0028	HP0032	Hypothetical	68	91	24
jhp0080	HP0087^d^	Hypothetical	89	96	8
jhp0173	HP0185^d^	Hypothetical	88	93	7
jhp0395	HP1029^d^	Hypothetical	88	95	7

^a ^The sequences of the indicated gene products in strain J99 were compared with corresponding sequences in each of the other 4 strains (26695, HPAG1, B128, and 98-10), and mean % amino acid identities were calculated.

^b ^The sequences of the indicated gene products in each strain were compared in all permutations, except that comparisons involving strain J99 were excluded from analysis. Mean % amino acid identities were calculated.

^c^Percentage of aligned sites in which the protein from strain J99 contained an amino acid different from the corresponding amino acids in proteins from 4 other strains.

^d ^Reported to be a constituent of the *H. pylori *core genome, based on at least one array analysis [18-20].

### Identification of novel strain-specific genes

To identify strain-specific genes uniquely present in one of the two newly sequenced genomes but not previously sequenced *H. pylori *genomes, we again used a BLAST score ratio analysis, as described in the Methods (Figure 2). Strain 98-10 contained 22 novel strain-specific genes and strain B128 contained 51 (Additional files 2 and 3). In addition, we identified 16 genes that were present in both strain 98-10 and B128, but not present in any of the previously sequenced strains (Additional file 4). Several of the strain-specific ORFs in *H. pylori *strains 98-10 and B128 were <100 nucleotides in length, and it is uncertain whether or not these very short ORFs are actually translated into proteins. An analysis of unique strain specific genes in the three previously sequenced *H. pylori *genomes (26695, J99, and HPAG1) revealed a similar number of unique strain-specific genes (Table 1), which have been described in previous studies [29-31].

To identify potential functions of the strain-specific genes found solely in strain 98-10 or B128 (or both 98-10 and B128), the deduced protein sequences were used as queries for BLAST searching of an NCBI database of non-redundant protein sequences (Table 4 and Additional files 2, 3, 4). Most of the strain-specific proteins found solely in strain 98-10 or B128 were not closely related to any known proteins or were related to proteins in the database for which the functions are not known. Several of the strain-specific genes found exclusively in strain 98-10 or B128 have been previously detected in strains of *H. pylori *for which the genome sequences have not been determined. As described above, insertion sequences and transposase-encoding genes (IS607 and ISHp608) were identified. Two strain-specific genes in *H. pylori *strain B128 (HPB128_11g15 and HPB128_11g23) encoded proteins related to type IV secretion system components (VirB9 and VirD4, respectively). The genes in this cluster (spanning HPB128_11g15 to HPB128_11g23) were not detected in the original genomic analyses of strains J99, 26695, or HPAG1 [29-31], but were subsequently detected in strain J99 and several other *H. pylori *strains [43].

**Table 4:** Strain-specific *H. pylori *genes present exclusively in strain 98-10 or B128

	Number of genes in the indicated strain(s)^a^		
	98-10	B128	98-10 and B128
Total number of strain-specific genes^a^	22	51	16
Functional class			
Transposase	2	3	6
Type IV secretion gene cluster^b^	0	7	0
Hypothetical	17	37	9
No database match	8	8	2
Closest match lacks known function	9	29	7
Other	3	4	1
Gene islands containing strain-specific genes^c^	2	11	3

^a^Present in the indicated strain(s), but not in any of the other four strains for which genome sequences are available.

^b^This group of genes was not detected in the original analysis of the genome from strain J99, but was subsequently detected in strain J99 [43].

^c^For this analysis, an island was considered to be present if two or more strain-specific genes were in contiguous chromosomal loci.

Interestingly, strain B128 contains several genes (HPB128_155g19, HPB128_156g11, HPB128_156g12, HPB128_184g1, HPB128_190g1) predicted to encode proteins that are more closely related to proteins encoded by *H. acinonychis *(a *Helicobacter *species isolated from large cats) [44] or *H. cetorum *(a *Helicobacter *species isolated from sea mammals) [45] than to any previously reported *H. pylori *protein sequences (Additional file 3). Similarly, strain 98-10 contains a gene (HP9810_5g6) predicted to encode a protein more closely related to a protein encoded by *H. cetorum *than to any previously reported *H. pylori *protein sequences (Additional file 2).

Subsets of the strain-specific genes found exclusively in strain 98-10 or B128 were found in contiguous chromosomal loci (Table 4). Two such gene clusters were identified in strain 98-10 and 11 were identified in strain B128. These gene clusters ranged from two to nine genes in length. Most of the gene clusters encode proteins of unknown function, but as noted above, one cluster encoded transposases and one cluster encoded two genes with homology to type IV secretion system components. The % G+C contents of three gene clusters in strain B128 (containing ORFs HPB128_65g16, HPB128_65g17, HPB128_156g11, HPB128_156g12, HPB128_192g1, HPB128_192g2, and HPB128_192g3, encoding proteins of unknown function) were each <30%, a value substantially lower than the total % G+C content of strain B128 (38.8%) and lower than the % G+C content of previously analyzed *H. pylori *strains (39%) [29,30]. The low % G+C content of these gene clusters suggests that these segments of DNA may have been acquired via horizontal transfer events.

### Strain-specific genes present in strains associated with gastric cancer or premalignant gastric lesions

Atrophic gastritis is a premalignant lesion [3], and *H. pylori*-infected patients with gastric ulcer disease have an increased risk of gastric cancer compared to *H. pylori*-infected patients with duodenal ulcer disease [46,47]. Strain 98-10 was isolated from a patient with gastric cancer, and strains HPAG1 and B128 were isolated from patients with atrophic gastritis and gastric ulcer disease, respectively. Therefore, we sought to identify strain-specific genes present in these three strains, but absent from the other two strains for which genome sequences were available (strains 26695 and J99, isolated from patients with superficial gastritis and duodenal ulcer disease, respectively). Ten strain-specific genes were found in the former 3 strains but were absent from strains 26695 and J99 (Table 5 and Additional file 5). These included 5 genes encoding restriction-modification systems and a gene predicted to encode an outer membrane protein; a previous study reported marked strain-specific variation in the size and sequence of this outer membrane protein [48]. We also performed a similar analysis to identify strain-specific genes present in various pairs of strains associated with malignant or premalignant conditions, but absent from strains 26695 and J99 (Table 5 and Additional files 4, 6, 7). The most commonly identified genes in these groups encoded restriction-modification systems or hypothetical proteins (Table 5 and Additional files 4, 6, 7). One of the genes in strains B128 and HPAG1 (*hrgA) *is a restriction endonuclease-replacing gene that was previously reported to be more prevalent among strains from Asian gastric cancer patients than among strains from non-cancer patients [49]. Three strain-specific genes found in strain B128 and strain HPAG1 (HPB128_146g1, HPB128_146g2, and HPB128_146g3) were previously reported to be localized on a plasmid in strain HPAG1 [31]. Another gene found exclusively in strain B128 and HPAG1 (HPB128_141g11) is found at the 3' end of the *cag *PAI in some *H. pylori *strains, and encodes a protein of unknown function (designated HP0521B) [50]. Strain-specific genes shared by 26695 and J99 (associated with superficial gastritis and duodenal ulcer disease), but not present in the three strains from patients with gastric cancer or premalignant gastric lesions are listed in Additional file 8.

**Table 5:** Strain-specific genes present exclusively in *H. pylori *strains associated with gastric cancer or premalignant lesions

	Number of genes in the indicated strains^a^			
	98-10, B128, and HPAG1	98-10 and B128	98-10 and HPAG1	B128 and HPAG1
Total number of strain-specific genes^a^	10	16	2	14
Functional class				
Restriction/modification	5	6	2	4
Hypothetical	3	9	0	4
No database match	0	2	0	0
Closest match lacks known function	3	7	0	4
Other	2	1	0	6

^a^Present in the indicated strains, but not in the other strains for which genome sequences are available.

## Discussion

In this study, we analyzed the genome sequences of an *H. pylori *strain isolated from a patient with gastric cancer and an *H. pylori *strain from a patient with gastric ulcer disease, and compared these with previously determined genome sequences of *H. pylori *strains associated with superficial gastritis, atrophic gastritis, and duodenal ulcer disease. We identified 1237 genes that were present in all 5 of these *H. pylori *strains. This group of genes may be considered to represent the *H. pylori *core genome. Some of the genes within the core genome are predicted to be essential for *H. pylori *viability *in vitro*. One previous study identified 33 genes that were essential for *H. pylori *viability [51]; all of these essential genes were present in the list of 1237 core genes identified in the current study. Other genes in the *H. pylori *core genome are not required for bacterial viability *in vitro*, but are predicted to be essential for *H. pylori *colonization of the stomach. Among 47 genes essential for *H. pylori *colonization of a gerbil model [52], 45 were included in the core genome described in the current study. Similarly, among 23 genes essential for *H. pylori *colonization of a mouse model (based on detection of a colonization defect in two different *H. pylori *strains) [53], 19 were included in the core genome described in the current study.

Several previous studies used array-based methodology to identify genes that are consistently present in all *H. pylori *strains analyzed [18-20]. The core genomes described in these previous studies have ranged from 1091 genes to 1281 genes. Potential reasons for differences in the reported size of the *H. pylori *core genome include variations in the number and choice of *H. pylori *strains selected for analysis, as well as variation in the DNA sequences that were used for array synthesis. In comparison to array-based methods, genome sequence analysis offers several potential advantages for delineation of a core genome. For example, genome sequence analysis is likely to be superior to array-based assays when differentiating between closely related paralogues, and genome sequence analysis is more likely to be successful in detecting the existence of highly divergent alleles. The main limitation of the sequence-based approach used in the current study for delineation of a core genome is that a relatively small number of genomes was analyzed. Nevertheless, there was reasonably close agreement between the core genes identified in the current study and the core genes identified in a previous array study [18].

Analysis of the 1237 core genes identified in this study revealed that the nucleotide sequences of these genes in individual strains were typically non-identical, and were differentiated by the presence of both synonymous and non-synonymous substitutions. As expected, allelic variation was detected within several housekeeping genes that have previously been used for MLST analysis. MLST analysis indicated that one of the strains analyzed in this study (98-10) belonged to an East Asian population cluster of *H. pylori *strains, whereas the other strains for which genome sequences were available belonged to European or West African population clusters. Thus, strain 98-10 is the first *H. pylori *strain from an East Asian population cluster to be analyzed by genome sequence analysis. We then focused on the identification of core genes in strain 98-10 that encoded proteins that were highly divergent compared to proteins encoded by the other 4 strains for which genome sequences were available. Eight such genes were identified (Table 2). Two of the genes shown in Table 2 (*cagA *and a *sel1 *homologue) have previously been reported to be highly divergent in East Asian strains compared to Western strains [13,42]. We speculate that several of the other genes listed in Table 2 may exhibit similar patterns of geographic divergence. Potentially the observed high level of divergence is associated with alterations in the functional activities of these proteins. The approach used in the current study prioritized identification of alleles that were highly divergent in one strain but similar in length compared to alleles in four other strains. A larger number of highly divergent alleles would have been identified if genes with substantial variations in length were included.

We identified several strain-specific genes in strain 98-10 or strain B128 that had not been previously described. Many of the new strain-specific genes identified in the current study were not closely related to any genes in the databases or were related to proteins for which the functions are not known. Notably, several of the new strain-specific genes identified in this study were closely related to genes present in related *Helicobacter *species, such as *H. acinonychis *[44] and *H. cetorum *[45].

Three of the strains for which genome sequences were available were isolated from patients with gastric cancer (98-10) or premalignant gastric lesions (atrophic gastritis and gastric ulcer; HPAG1 and B128). Therefore, we sought to identify genes present in these strains that were absent from strains isolated from patients with non-malignant conditions. We identified numerous genes that fulfilled these criteria (Table 5). Potentially several of these may be useful biomarkers for strains capable of inducing malignant or premalignant gastric lesions. Further studies involving larger numbers of strains will be needed in order to test this hypothesis.

Finally, it is notable that one of the strains selected for analysis in the current study (strain 98-10) was isolated from a gastric cancer patient in Japan, a country with a very high incidence of gastric cancer [3,35]. The biological basis for geographic variation in the incidence of gastric cancer is not yet clearly understood. Both environmental factors (such as a high-salt diet) and host genetic factors may be contributory [2,3]. In addition, *H. pylori *strains circulating in some parts of the world may have an increased carcinogenic potential compared to strains circulating in other parts of the world. In support of the latter hypothesis, most *H. pylori *strains isolated in Japan express forms of CagA that have multiple sites where tyrosine phosphorylation can occur and a unique tyrosine phosphorylation site (EPIYA-D), resulting in high levels of tyrosine-phosphorylated CagA within gastric epithelial cells and potent activation of the SHP-2 tyrosine phosphatase [13,14,25]. In future studies, it will be important to study further the geographic variations that exist among *H. pylori *genomes by analyzing a larger number of strains, and to determine whether the presence of particular allelic variations or strain-specific genes correlates with specific disease outcomes such as gastric cancer.

## Conclusion

In this study we analyzed the genome sequences of an *H. pylori *strain isolated from a patient with gastric cancer and a strain isolated from a patient with gastric ulcer disease. Each strain contained novel genes not present in previously described *H. pylori *genomes. In addition, highly divergent alleles were identified. Comparative analysis of *H. pylori *strains isolated from patients with different clinical conditions provides a foundation for understanding why *H. pylori *may be associated with a variety of different gastroduodenal diseases.

## Methods

### *H. pylori *strains

*H. pylori *strain 98-10 was isolated from a patient in Japan with gastric adenocarcinoma [34]. *H. pylori *strain B128 was isolated from a patient in the United States with a gastric ulcer [32]. The genome sequences of *H. pylori *strains 26695, J99, and HPAG1 have been published previously [29-31]. Strain 26695 was isolated from a patient in the United Kingdom with gastritis [29]. Strain J99 was isolated from a patient in the United States with duodenal ulcer disease [30]. Strain HPAG1 was isolated from a patient in Sweden with chronic atrophic gastritis [31].

### Genome sequencing

A single colony of *H. pylori *98-10 and a single colony of strain B128 were isolated and DNA was purified as described previously [54]. DNA sequencing was accomplished using an emulsion method for DNA amplification, and an instrument (Genome Sequence 20 System) that performs pyrophosphate-based sequencing (pyrosequencing) in picolitre-sized wells (454 Life Sciences, Branford, CT). Random libraries of DNA fragments were generated by shearing an entire genome and isolating single DNA molecules by limiting dilution. Specialized common adapters were added to the fragments, the individual fragments were captured on their own beads and, within the droplets of an emulsion, the individual fragments were clonally amplified [55]. This approach does not require subcloning in bacteria or the handling of individual clones, as the templates were handled in bulk within the emulsions. Three runs of the sequencing instrument were used for analysis of strain 98-10 and two instrument runs were used for analysis of strain B128. Assembly of sequence data was performed as described by Margulies et al. [55]. The average depth of sequencing coverage was approximately 20-fold. Sequence data from strain 98-10 were assembled into 51 large contigs, each > 600 nucleotides in size (average contig length 30,819 nucleotides). Sequence data from strain B128 were assembled into 73 large contigs, each > 600 nucleotides in size (average contig length 22,592 nucleotides). As described by Oh et al. [31], analysis of an *H. pylori *genome via this approach yields results comparable to results obtained by traditional Sanger sequencing.

### Analysis of sequence data

ORFs in the genomes of *H. pylori *strains 98-10 and B128 were predicted by FGENESB [56], an algorithm based on Markov chain models of coding regions and translation and termination sites that was "trained" on the genome from *H. pylori *strain 26695.

### Multi-locus sequence typing

To analyze relationships between the strains analyzed in this study and other globally distributed *H. pylori *patient isolates, we used a multilocus sequence typing (MLST) database  containing data on 434 *H. pylori *strains that were isolated from patients in a broad range of geographic locations. This MLST database contains sequence data (398 to 627 bp per gene) for eight core genes *(atpA, efp, mutY, ppa, trpC, ureI, vacA, and yphC) *that are distributed throughout the *H. pylori *genome. Nucleotide sequences of the concatenated MLST loci were aligned using ClustalW algorithm within MEGA4. Phylogenetic relationships were constructed using MEGA4 with the Kimura 2-parameter model of nucleotide substitution and neighbor-joining clustering [57,58]. The tree shown in Figure 1 is the product of 1000 bootstrap replicates [59].

### Identification of strain-specific genes and core genes

To identify strain-specific genes and genes present in all 5 *H. pylori *genomes analyzed in the current study, we used a BLAST score ratio (BSR) algorithm [60]. This algorithm is based on an analysis of BLAST raw scores, which, in contrast to comparison to analysis of BLAST output E-values, more accurately accounts for the length of the similarity between the Reference and Query sequences. As a first step, ORFs were translated into deduced amino acid sequences. BLAST score ratios were computed by first determining the BLAST raw score for each Reference peptide against itself; this raw score was designated as the Reference score. Each Reference peptide was then compared to each peptide in individual query proteomes, and each best BLAST raw score was recorded. The BSR was calculated by dividing the Query score by the Reference score for each Reference peptide. Thus, all BSRs were normalized within a range between 0 and 1. A score of 1 indicates a perfect match of the Reference peptide to a Query peptide and a score of 0 indicates no BLAST match of the Reference peptide in the Query proteome. To identify strain-specific genes, multiple separate analyses were performed, each using a different strain as the reference. A BSR threshold value of 0.4 was used for identification of strain-specific genes. This stringent threshold value corresponds to approximately 30% amino acid identity over approximately 30% of the peptide length, a commonly used threshold for peptide similarity [60]. The same analytical approach was used to identify core genes that were present in all 5 strains for which genome sequences were available.

### Identification and analysis of alleles encoding highly divergent gene products

Among the core genes that were identified in all five *H. pylori *strains (BSR >0.4), we sought to identify alleles found in a single strain that differed markedly from corresponding alleles found in the other four strains. Candidate divergent alleles in a particular strain were initially identified by selecting peptides having a 0.4<BSR<0.9 in multiple analyses, each using a different strain as the reference. Deduced amino acid sequences from the 5 strains were aligned and compared using the NWay Comp program [61]. Alignments were manually inspected to exclude cases in which low BSRs were primarily attributable to differences in peptide length. Each gene product of interest from strain A was compared with corresponding gene products from strains B, C, D, and E, and a mean % amino acid identity value was calculated; similarly, the gene products in strains B, C, D, and E were compared in all permutations, and a mean % amino acid identity value was calculated. A gene from strain A was considered highly divergent if the former value was significantly lower than the latter value.

### Sequence data

This Whole Genome Shotgun project has been deposited at DDBJ/EMBL/GenBank under the project accession ABSX00000000 (for strain 98-10) and ABSY00000000 (for strain B128). The versions described in this paper are the first versions (ABSX01000000 and ABSY01000000)

## Authors' contributions

MM participated in the design of this study, analyzed genome sequences, and helped to draft the manuscript. CS performed the MLST analysis and helped to draft the manuscript. DI and RP contributed genome sequence data for strain B128. TC helped to design the study, analyzed genome sequences, and helped to draft the manuscript. All authors read and approved the final manuscript.

## Supplementary Material

Additional File 1 **Genes present in all five of the *H. pylori *strains analyzed**. This file contains a list of genes that are present in all five of the *H. pylori *strains analyzed (26695, J99, HPAG1, 98-10, and B128). Click here for file

Additional File 2 **Genes present exclusively in *H. pylori *strain 98-10**. This file contains a list of genes that are present in strain 98-10 (from a Japanese patient with gastric cancer), but not in the other 4 strains analyzed. Click here for file

Additional File 3 **Genes present exclusively in *H. pylori *strain B128**. This file contains a list of genes that are present in strain B128 (from a patient with gastric ulcer disease), but not in the other 4 strains analyzed. Click here for file

Additional File 4 **Genes present exclusively in *H. pylori *strains 98-10 and B128**. This file contains a list of genes that are present in strains 98-10 and B128 (from patients with gastric cancer and gastric ulcer, respectively), but not in the three other strains analyzed. Click here for file

Additional File 5 **Genes present exclusively in *H. pylori *strains 98-10, B128, and HPAG1**. This file contains a list of genes that are present in strains from patients with gastric cancer or premalignant conditions, but not in the two other strains analyzed. Click here for file

Additional File 6 **Genes present exclusively in *H. pylori *strains 98-10 and HPAG1**. This file contains a list of genes that are present in strains 98-10 and HPAG1 (from patients with gastric cancer and atrophic gastritis), but not in the other 3 strains analyzed. Click here for file

Additional File 7 **Genes present exclusively in *H. pylori *strains B128 and HPAG1**. This file contains a list of genes that are present in strains B128 and HPAG1 (from patients with gastric ulcer and atrophic gastritis), but not in the other 3 strains analyzed. Click here for file

Additional File 8 **Genes present exclusively in *H. pylori *strains 26695 and J99**. This file contains a list of genes that are present in strains 26695 and J99 (from patients with superficial gastritis and duodenal ulcer), but not in the 3 strains from patients with gastric cancer or premalignant conditions. Click here for file

## References

[B1] Suerbaum S, Michetti P (2002). *Helicobacter pylori *infection. N Engl J Med.

[B2] Peek RM, Blaser MJ (2002). *Helicobacter pylori *and gastrointestinal tract adenocarcinomas. Nat Rev Cancer.

[B3] Fuchs CS, Mayer RJ (1995). Gastric carcinoma. N Engl J Med.

[B4] Blaser MJ, Berg DE (2001). *Helicobacter pylori *genetic diversity and risk of human disease. J Clin Invest.

[B5] Linz B, Schuster SC (2007). Genomic diversity in *Helicobacter *and related organisms. Res Microbiol.

[B6] Suerbaum S, Smith JM, Bapumia K, Morelli G, Smith NH, Kunstmann E, Dyrek I, Achtman M (1998). Free recombination within *Helicobacter pylori*. Proc Natl Acad Sci USA.

[B7] Suerbaum S, Josenhans C (2007). *Helicobacter pylori *evolution and phenotypic diversification in a changing host. Nat Rev Microbiol.

[B8] Atherton JC, Cao P, Peek RM, Tummuru MK, Blaser MJ, Cover TL (1995). Mosaicism in vacuolating cytotoxin alleles of *Helicobacter pylori*. Association of specific *vacA *types with cytotoxin production and peptic ulceration. J Biol Chem.

[B9] Cao P, Cover TL (2002). Two different families of *hopQ *alleles in *Helicobacter pylori*. J Clin Microbiol.

[B10] Achtman M, Azuma T, Berg DE, Ito Y, Morelli G, Pan ZJ, Suerbaum S, Thompson SA, Ende A van der, van Doorn LJ (1999). Recombination and clonal groupings within *Helicobacter pylori *from different geographical regions. Mol Microbiol.

[B11] Wirth T, Wang X, Linz B, Novick RP, Lum JK, Blaser M, Morelli G, Falush D, Achtman M (2004). Distinguishing human ethnic groups by means of sequences from *Helicobacter pylori *: lessons from Ladakh. Proc Natl Acad Sci USA.

[B12] Linz B, Balloux F, Moodley Y, Manica A, Liu H, Roumagnac P, Falush D, Stamer C, Prugnolle F, Merwe SW van der, Yamaoka Y, Graham DY, Perez-Trallero E, Wadstrom T, Suerbaum S, Achtman M (2007). An African origin for the intimate association between humans and *Helicobacter pylori*. Nature.

[B13] Higashi H, Tsutsumi R, Fujita A, Yamazaki S, Asaka M, Azuma T, Hatakeyama M (2002). Biological activity of the *Helicobacter pylori *virulence factor CagA is determined by variation in the tyrosine phosphorylation sites. Proc Natl Acad Sci USA.

[B14] Hatakeyama M (2004). Oncogenic mechanisms of the *Helicobacter pylori *CagA protein. Nat Rev Cancer.

[B15] Van Doorn LJ, Figueiredo C, Megraud F, Pena S, Midolo P, Queiroz DM, Carneiro F, Vanderborght B, Pegado MD, Sanna R, De Boer W, Schneeberger PM, Correa P, Ng EK, Atherton J, Blaser MJ, Quint WG (1999). Geographic distribution of *vacA *allelic types of *Helicobacter pylori*. Gastroenterology.

[B16] Devi SM, Ahmed I, Francalacci P, Hussain MA, Akhter Y, Alvi A, Sechi LA, Megraud F, Ahmed N (2007). Ancestral European roots of *Helicobacter pylori *in India. BMC Genomics.

[B17] Falush D, Wirth T, Linz B, Pritchard JK, Stephens M, Kidd M, Blaser MJ, Graham DY, Vacher S, Perez-Perez GI, Yamaoka Y, Megraud F, Otto K, Reichard U, Katzowitsch E, Wang X, Achtman M, Suerbaum S (2003). Traces of human migrations in *Helicobacter pylori *populations. Science.

[B18] Gressmann H, Linz B, Ghai R, Pleissner KP, Schlapbach R, Yamaoka Y, Kraft C, Suerbaum S, Meyer TF, Achtman M (2005). Gain and loss of multiple genes during the evolution of *Helicobacter pylori*. PLoS Genet.

[B19] Salama N, Guillemin K, McDaniel TK, Sherlock G, Tompkins L, Falkow S (2000). A whole-genome microarray reveals genetic diversity among *Helicobacter pylori *strains. Proc Natl Acad Sci USA.

[B20] Han YH, Liu WZ, Shi YZ, Lu LQ, Xiao S, Zhang QH, Zhao GP (2007). Comparative genomics profiling of clinical isolates of *Helicobacter pylori *in Chinese populations using DNA microarray. J Microbiol.

[B21] Figueiredo C, Machado JC, Pharoah P, Seruca R, Sousa S, Carvalho R, Capelinha AF, Quint W, Caldas C, van Doorn LJ, Carneiro F, Sobrinho-Simoes M (2002). *Helicobacter pylori *and interleukin 1 genotyping: an opportunity to identify high-risk individuals for gastric carcinoma. J Natl Cancer Inst.

[B22] Gerhard M, Lehn N, Neumayer N, Boren T, Rad R, Schepp W, Miehlke S, Classen M, Prinz C (1999). Clinical relevance of the *Helicobacter pylori *gene for blood-group antigen-binding adhesin. Proc Natl Acad Sci USA.

[B23] Dossumbekova A, Prinz C, Mages J, Lang R, Kusters JG, van Vliet AHM, Reindl W, Backert S, Saur D, Schmid RM, Rad R (2006). *Helicobacter pylori *HopH (OipA) and bacterial pathogenicity: genetic and functional genomic analysis of *hopH *gene polymorphisms. J Infect Dis.

[B24] Censini S, Lange C, Xiang Z, Crabtree JE, Ghiara P, Borodovsky M, Rappuoli R, Covacci A (1996). *cag*, a pathogenicity island of *Helicobacter pylori*, encodes type I-specific and disease-associated virulence factors. Proc Natl Acad Sci.

[B25] Maeda S, Ogura K, Yoshida H, Kanai F, Ikenoue T, Kato N, Shiratori Y, Omata M (1998). Major virulence factors, VacA and CagA, are commonly positive in *Helicobacter pylori *isolates in Japan. Gut.

[B26] Maeda S, Yoshida H, Ikenoue T, Ogura K, Kanai F, Kato N, Shiratori Y, Omata M (1999). Structure of *cag *pathogenicity island in Japanese *Helicobacter pylori *isolates. Gut.

[B27] Bourzac KM, Guillemin K (2005). *Helicobacter pylori*-host cell interactions mediated by type IV secretion. Cell Microbiol.

[B28] Blaser MJ, Perez-Perez GI, Kleanthous H, Cover TL, Peek RM, Chyou PH, Stemmermann GN, Nomura A (1995). Infection with *Helicobacter pylori *strains possessing cagA is associated with an increased risk of developing adenocarcinoma of the stomach. Cancer Res.

[B29] Tomb J-F, White O, Kerlavage AR, Clayton RA, Sutton GG, Fleischmann RD, Ketchum KA, Klenk HP, Gill S, Dougherty BA, Nelson K, Quackenbush J, Zhou L, Kirkness EF, Peterson S, Loftus B, Richardson D, Dodson R, Khalak HG, Glodek A, McKenney K, Fitzegerald LM, Lee N, Adams MD, Hickey EK, Berg DE, Gocayne JD, Utterback TR, Peterson JD, Kelley JM, Cotton MD, Weidman JM, Fujii C, Bowman C, Watthey L, Wallin E, Hayes WS, Borodovsky M, Karp PD, Smith HO, Frazer CM, Venter JC (1997). The complete genome sequence of the gastric pathogen *Helicobacter pylori*. Nature.

[B30] Alm RA, Ling LS, Moir DT, King BL, Brown ED, Doig PC, Smith DR, Noonan B, Guild BC, deJonge BL, Carmel G, Tummino PJ, Caruso A, Uria-Nickelsen M, Mills DM, Ives C, Gibson R, Merberg D, Mills SD, Jiang Q, Taylor DE, Vovis GF, Trust TJ (1999). Genomic-sequence comparison of two unrelated isolates of the human gastric pathogen *Helicobacter pylori*. Nature.

[B31] Oh JD, Kling-Backhed H, Giannakis M, Xu J, Fulton RS, Fulton LA, Cordum HS, Wang C, Elliott G, Edwards J, Mardis ER, Engstrand LG, Gordon JI (2006). The complete genome sequence of a chronic atrophic gastritis *Helicobacter pylori *strain: evolution during disease progression. Proc Natl Acad Sci USA.

[B32] Israel DA, Salama N, Arnold CN, Moss SF, Ando T, Wirth HP, Tham KT, Camorlinga M, Blaser MJ, Falkow S, Peek RM (2001). *Helicobacter pylori *strain-specific differences in genetic content, identified by microarray, influence host inflammatory responses. J Clin Invest.

[B33] Fox JG, Wang TC, Rogers AB, Poutahidis T, Ge Z, Taylor N, Dangler CA, Israel DA, Krishna U, Gaus K, Peek RM (2003). Host and microbial constituents influence *Helicobacter pylori*-induced cancer in a murine model of hypergastrinemia. Gastroenterology.

[B34] Ando T, Peek RM, Pride D, Levine SM, Takata T, Lee YC, Kusugami K, Ende A van der, Kuipers EJ, Kusters JG, Blaser MJ (2002). Polymorphisms of *Helicobacter pylori *HP0638 reflect geographic origin and correlate with cagA status. J Clin Microbiol.

[B35] Leung WK, Wu MS, Kakugawa Y, Kim JJ, Yeoh KG, Goh KL, Wu KC, Wu DC, Sollano J, Kachintorn U, Gotoda T, Lin JT, You WC, Ng EK, Sung JJ, Asia Pacific Working Group on Gastric Cancer (2008). Screening for gastric cancer in Asia: current evidence and practice. Lancet Oncol.

[B36] Kersulyte D, Mukhopadhyay AK, Shirai M, Nakazawa T, Berg DE (2000). Functional organization and insertion specificity of IS607, a chimeric element of *Helicobacter pylori*. J Bacteriol.

[B37] Kersulyte D, Velapatino B, Dailide G, Mukhopadhyay AK, Ito Y, Cahuayme L, Parkinson AJ, Gilman RH, Berg DE (2002). Transposable element ISHp608 of *Helicobacter pylori *: nonrandom geographic distribution, functional organization, and insertion specificity. J Bacteriol.

[B38] Cover TL, Blanke SR (2005). *Helicobacter pylori *VacA, a paradigm for toxin multifunctionality. Nat Rev Microbiol.

[B39] Busler VJ, Torres VJ, McClain MS, Tirado O, Friedman DB, Cover TL (2006). Protein-protein interactions among *Helicobacter pylori *Cag proteins. J Bacteriol.

[B40] van Doorn LJ, Figueiredo C, Sanna R, Pena S, Midolo P, Ng EK, Atherton JC, Blaser MJ, Quint WG (1998). Expanding allelic diversity of *Helicobacter pylori vacA*. J Clin Microbiol.

[B41] Franco AT, Johnston E, Krishna U, Yamaoka Y, Israel DA, Nagy TA, Wroblewski LE, Piazuelo MB, Correa P, Peer RM (2008). Regulation of gastric carcinogenesis by *Helicobacter pylori *virulence factors. Cancer Res.

[B42] Ogura M, Perez JC, Mittl PR, Lee HK, Dailide G, Tan S, Ito Y, Secka O, Dailidiene D, Putty K, Berg DE, Kalia A (2007). *Helicobacter pylori *evolution: lineage- specific adaptations in homologs of eukaryotic Sel1-like genes. PLoS Comput Biol.

[B43] Kersulyte D, Velapatino B, Mukhopadhyay AK, Cahuayme L, Bussalleu A, Combe J, Gilman RH, Berg DE (2003). Cluster of type IV secretion genes in *Helicobacter pylori's *plasticity zone. J Bacteriol.

[B44] Eppinger M, Baar C, Linz B, Raddatz G, Lanz C, Keller H, Morelli G, Gressmann H, Achtman M, Schuster SC (2006). Who ate whom? Adaptive *Helicobacter *genomic changes that accompanied a host jump from early humans to large felines. PLoS Genet.

[B45] Harper CG, Feng Y, Xu S, Taylor NS, Kinsel M, Dewhirst FE, Paster BJ, Greenwell M, Levine G, Rogers A, Fox JG (2002). *Helicobacter cetorum *sp. nov., a urease-positive *Helicobacter *species isolated from dolphins and whales. J Clin Microbiol.

[B46] Hansson LE, Nyren O, Hsing AW, Bergstrom R, Josefsson S, Chow WH, Fraumeni JF, Adami HO (1996). The risk of stomach cancer in patients with gastric or duodenal ulcer disease. N Engl J Med.

[B47] Parsonnet J (1996). *Helicobacter pylori *in the stomach–a paradox unmasked. N Engl J Med.

[B48] Sumie A, Yamashiro T, Nakashima K, Nasu M, Watanabe M, Nishizono A (2001). Comparison of genomic structures and antigenic reactivities of orthologous 29-kilodalton outer membrane proteins of *Helicobacter pylori*. Infect Immun.

[B49] Ando T, Wassenaar TM, Peek RM, Aras RA, Tschumi AI, van Doorn LJ, Kusugami K, Blaser MJ (2002). A *Helicobacter pylori *restriction endonuclease-replacing gene, *hrgA*, is associated with gastric cancer in Asian strains. Cancer Res.

[B50] Blomstergren A, Lundin A, Nilsson C, Engstrand L, Lundeberg J (2004). Comparative analysis of the complete *cag *pathogenicity island sequence in four *Helicobacter pylori *isolates. Gene.

[B51] Chalker AF, Minehart HW, Hughes NJ, Koretke KK, Lonetto MA, Brinkman KK, Warren PV, Lupas A, Stanhope MJ, Brown JR, Hoffman PS (2001). Systematic identification of selective essential genes in *Helicobacter pylori *by genome prioritization and allelic replacement mutagenesis. J Bacteriol.

[B52] Kavermann H, Burns BP, Angermuller K, Odenbriet S, Fischer W, Melchers K, Haas R (2003). Identification and characterization of *Helicobacter pylori *genes essential for gastric colonization. J Exp Med.

[B53] Baldwin DN, Shepherd B, Kraemer P, Hall MK, Sycuro LK, Pinto-Santini DM, Salama NR (2007). Identification of *Helicobacter pylori *genes that contribute to stomach colonization. Infect Immun.

[B54] Cover TL, Tummuru MKR, Cao P, Thompson SA, Blaser MJ (1994). Divergence of genetic sequences for the vacuolating cytotoxin among *Helicobacter pylori *strains. J Biol Chem.

[B55] Margulies M, Egholm M, Altman WE, Attiya S, Bader JS, Bemben LA, Berka J, Braverman MS, Chen YJ, Chen Z, Dewell SB, Du L, Fierro JM, Gomes XV, Godwin BC, He W, Helgesen S, Ho CH, Irzyk GP, Jando SC, Alenquer ML, Jarvie TP, Jirage KB, Kim JB, Knight JR, Lanza JR, Leamon JH, Lefkowitz SM, Lei M, Li J, Lohman KL, Lu H, Makhijani VB, McDade KE, McKenna MP, Myers EW, Nickerson E, Nobile JR, Plant R, Puc BP, Ronan MT, Roth GT, Sarkis GJ, Simons FJ, Simpson JW, Srinivasan M, Tartaro KR, Tomasz A, Vogt KA, Volkmer GA, Wang SH, Wang Y, Weiner MP, Yu P, Begley RF, Rothberg JM (2005). Genome sequencing in microfabricated high-density picolitre reactors. Nature.

[B56] Tyson GW, Chapman J, Hugenholtz P, Allen EE, Ram RJ, Richardson PM, Solovyev VV, Rubin EM, Rokhsar DS, Banfield JF (2004). Community structure and metabolism through reconstruction of microbial genomes from the environment. Nature.

[B57] Kimura M (1980). A simple method for estimating evolutionary rates of base substitutions through comparative studies of nucleotide sequences. J Mol Evol.

[B58] Saitou N, Nei M (1987). The neighbor-joining method: a new method for reconstructing phylogenetic trees. Mol Biol Evol.

[B59] Felsenstein J (1985). Confidence limits on phylogenies: an approach using the bootstrap. Evolution.

[B60] Rasko DA, Myers GS, Ravel J (2005). Visualization of comparative genomic analyses by BLAST score ratio. BMC Bioinformatics.

[B61] Yao J, Lin H, Doddapaneni H, Civerolo EL (2007). nWayComp: a genome-wide sequence comparison tool for multiple strains/species of phylogenetically related microorganisms. In Silico Biol.

[B62] Jolley KA, Chan MS, Maiden MC (2004). mlstdbNet – distributed multi-locus sequence typing (MLST) databases. BMC Bioinformatics.

[B63] Tamura K, Dudley J, Nei M, Kumar S (2007). MEGA4: Molecular Evolutionary Genetics Analysis (MEGA) software version 4.0. Mol Biol Evol.

